# Spontaneous coronary artery dissection in an underrepresented region: insights from the Serbian (RS) SCAD registry

**DOI:** 10.3389/fcvm.2026.1795347

**Published:** 2026-05-11

**Authors:** Svetlana Apostolovic, Dragana Stanojevic, Zlatko Mehmedbegovic, Gordana Krljanac, Ivan Ilic, Milovan Petrovic, Aleksandra Djokovic, Vladimir Mitov, Vladimir Zdravkovic, Mila Kovacevic, Bojan Maricic, Marija Andjelkovic Apostolovic, Miroslav Nikolic, Milenko Cankovic, Dragana Dabovic, Zeljko Zivanovic, Bojan Stanetic, Ivana Iveljic, Dusan Nikolic, Srdjan Aleksandric, Branko Beleslin

**Affiliations:** 1 Medical Faculty, University of Nis, Nis, Serbia; 2 Cardiology Clinic, University Clinical Center Nis, Nis, Serbia; 3 Faculty of Medicine, University of Belgrade, Belgrade, Serbia; 4 Cardiology Clinic, University Clinical Center Serbia, Belgrade, Serbia; 5 School of Medicine, University of Belgrade, Belgrade, Serbia; 6 Institute for Cardiovascular Diseases, Dedinje, Serbia; 7 Faculty of Medicine, University of Novi Sad, Novi Sad, Serbia; 8 Institute of Cardiovascular Diseases of Vojvodina, Cardiology Clinic, Sremska Kamenica, Serbia; 9 Cardiology Department, University Hospital Medical Center Bezanijska Kosa, Belgrade, Serbia; 10 Department of Interventional Cardiology, Medical Center Zajecar, Zajecar, Serbia; 11 Faculty of Medicine, University of Kragujevac, Kragujevac, Serbia; 12 Cardiology Clinic, University Clinical Center Kragujevac, Kragujevac, Serbia; 13 Medical Faculty, Department of Medical Statistics and Informatics, University of Nis, Niš, Serbia; 14 Center for Health Informatics and Biostatistics, Institute of Public Health Nis, Niš, Serbia; 15 Cardiology Clinic, University Clinical Centre of the Republic of Srpska, Banja Luka, Bosnia and Herzegovina; 16 Faculty of Medicine, University of Banja Luka, Banja Luka, Bosnia and Herzegovina; 17 Clinic for Invasive Cardiology, University Clinical Center Tuzla, Tuzla, Bosnia and Herzegovina

**Keywords:** acute coronary syndrome (ACS), clinical outcomes, coronary artery disease (CAD), registry study, spontaneous coronary artery dissection (SCAD)

## Abstract

**Background:**

Evidence on spontaneous coronary artery dissection (SCAD) is largely derived from Western registries, with limited data from Eastern transitional regions. The Serbian SCAD Registry provides the first comprehensive multicenter data from this region.

**Methods:**

Patients with angiographically confirmed SCAD were enrolled from 14 PCI centers and followed during hospitalization, at 30 days, and at 1 year.

**Results:**

Among 123 patients (mean age 47.8 ± 11.8 years; 85.4% female), traditional cardiovascular risk factors were highly prevalent (hypertension 49.6%, dyslipidemia 46.3%, smoking 41.5%), exceeding rates reported in Western cohorts. Emotional stress was the most common precipitating factor (38.5%). Left anterior descending artery involvement occurred in 56.1%, with Type 2a SCAD being the most frequent (42.1%). PCI was performed in 51.3%, and dual antiplatelet therapy was prescribed in 87%, both substantially higher than in Western registries.

In-hospital MACE occurred in 23.6% (mortality 8.1%). At 30 days, minor adverse events were observed, while one-year MACE was 8.2%.

**Conclusions:**

This Eastern European SCAD cohort demonstrates a distinct profile characterized by a high burden of traditional cardiovascular risk factors, frequent PCI use, and elevated in-hospital adverse events compared with Western registries. Greater adherence to guideline-based conservative management may improve outcomes.

## Introduction

Spontaneous coronary dissection (SCAD) is increasingly recognized as a critical etiology for acute coronary syndromes, especially among younger individuals and women (1, 2). This condition involves a spontaneous separation of the coronary artery wall layers, leading to an intramural hematoma that obstructs coronary blood flow (3).

SCAD accounts for 1.7%–4% of all acute coronary syndrome presentations, making it a notable cause of myocardial infarction and sudden cardiac death (2, 4, 5). It predominantly affects middle-aged women and individuals without conventional atherosclerotic risk factors (6, 7). The exact cause of SCAD remains largely unknown. However, the over-representation of young and middle-aged women, coupled with their association with pregnancy and postpartum periods, strongly suggests a potential pathophysiological role for female sex hormones (8).

The most common risk factors include exogenous hormone use, significant physical or emotional stressors, and underlying inflammatory or connective tissue disorders (9). The newer literature indicates that hypertension (18%–57%), hyperlipidemia (10%–52%), and smoking (11%–57%) are frequently observed, challenging the conventional paradigm of SCAD primarily afflicting healthy women (8).

However, despite its growing recognition, significant knowledge gaps persist regarding optimal management strategies and long-term outcomes, prompting the establishment of dedicated registries (10). Currently, all consensus documents on the diagnosis and treatment of SCAD are derived from individual case series and observational registries conducted in high-income Western countries (11). We present findings from the only SCAD registry in Eastern transitional countries—the Serbian (RS) SCAD Registry—which includes 123 patients from 14 PCI centers. This registry aims to elucidate the demographic, clinical, and angiographic characteristics of SCAD within the Serbian population. Also, we wanted to determine the short- and long-term outcomes in those patients and the prognostic indicators.

## Methods

The Serbian (RS) SCAD Registry is a multicenter, observational cohort study that is both prospective and retrospective. The Cardiology Society of Serbia, with its ethical committee, approved the survey in October 2021 (UKS registry number 26858).

The national registry enrolls patients with SCAD from 14 catheterization laboratories across Serbia, as well as from the Republic of Srpska and Bosnia and Herzegovina, that are affiliated with the Cardiology Society of Serbia (University Clinical Center, Republic of Srpska, Banja Luka, and University Clinical Center, Tuzla, Bosnia and Herzegovina). The local Ethics Committees of all included centers granted ethics approval.

**Figure 1:**
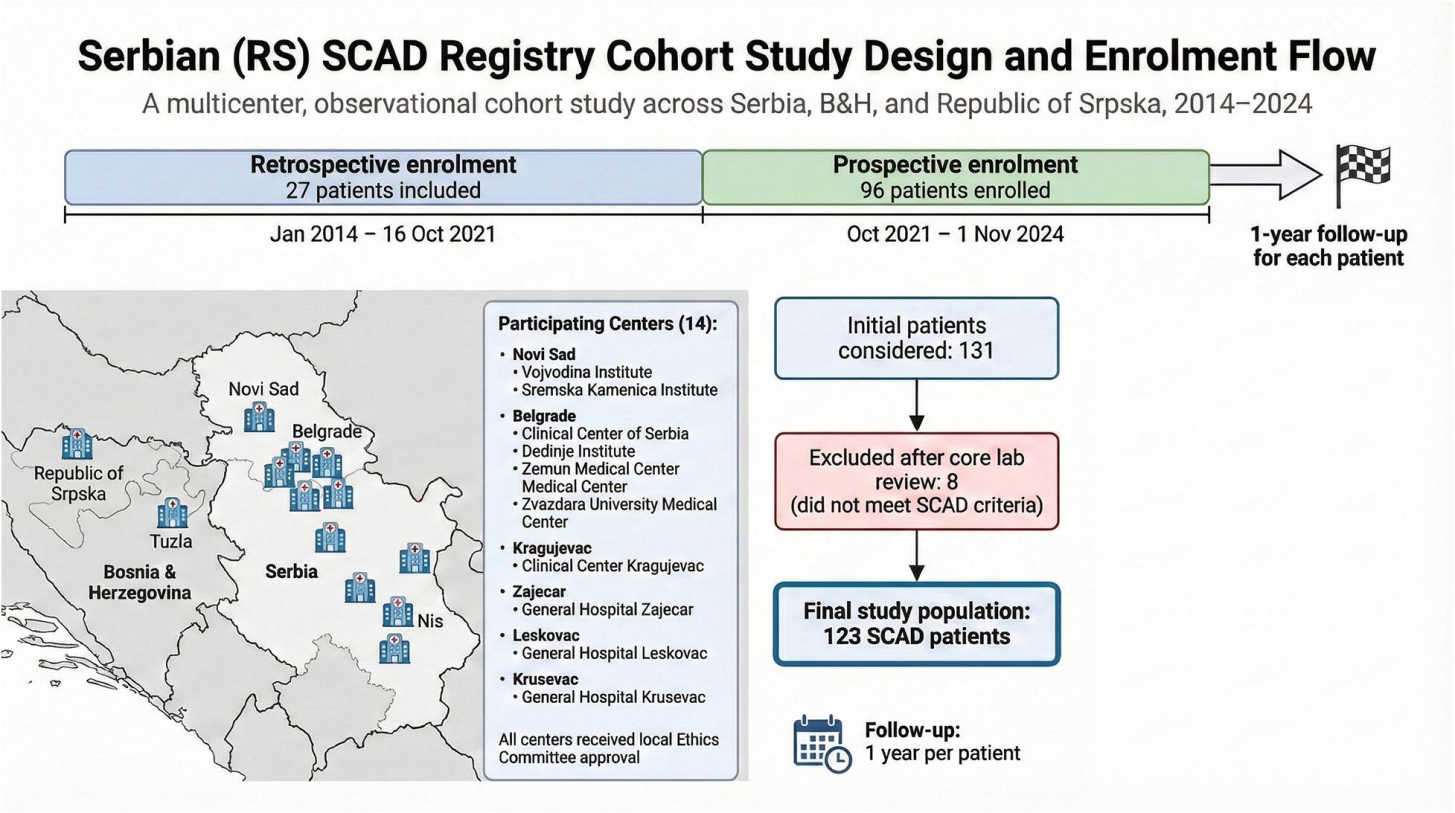
Flow chart for Serbian (RS) SCAD registry.

Besides the above-mentioned centers out of the territory of the Republic of Serbia in the multicenter study were included: the University Clinical Center Nis; the University Clinical Center of Vojvodina in Novi Sad; the University Clinical Center Belgrade; the University Clinical Center Kragujevac; the Clinical Hospital Center Zvezdara, Belgrade; Clinical Hospital Center Zemun, Belgrade; the Institute for Cardiovascular Diseases “Dedinje”, Belgrade; University Hospital Medical Center Bezanijska Kosa, Belgrade; and the Health Center Zajecar; Military Medical Academy Serbia-Clinic for Cardiology, Belgrade; General Hospital Leskovac and General Hospital Krusevac. Our multicenter observational study initially included 131 patients. Still, after careful review by our Core Lab, eight patients did not meet the criteria for SCAD. Our database has a retrospective and a prospective part. The former meticulously documents cases from January 2014 to 16th October 2021, while the latter encompasses a thorough examination of patient records spanning from October 2021 to 1st November 2024. Patients were followed for one year (Figure 1). This comprehensive approach enables robust analysis of SCAD trends and outcomes within the region, offering unique insights into a previously understudied demographic (3).

Patients recruited prospectively provided informed consent during the initial hospitalization, whereas those recruited retrospectively received a waiver of consent, in accordance with the Helsinki Declaration and local ethical committee requirements.

### Data collection

Data were collected from medical records for all recruited patients, including baseline and presenting characteristics, past medical history, in-hospital diagnostic procedures, treatments, and outcomes. For the prospective arm, patients were called for a control physical exam at 30 days and 1 year after the initial event. Telephone follow-up was conducted by the study coordinator on an occasional basis to check the patient's status. In the retrospective arm, the last date of contact and outcomes were taken from the available medical records at the time of recruitment.

Only patients with confirmed SCAD, as determined by independent core laboratory review of invasive coronary angiography, were included. Participating sites uploaded coronary angiography images to a central electronic image storage system, a secure online database hosted by the University Clinical Center Nis. Core laboratory adjudication was performed by at least one senior interventional cardiologist from each included PCI center. All other data were manually inserted into the secured electronic database. The angiographic Yip-Saw classification was used to classify SCAD. Type 1 SCAD is characterized by the presence of the false lumen with dual-lumen appearance, frequently accompanied by localized extraluminal dye residua following contrast clearance. This type of SCAD is pathophysiologically distinct from types 2 and 3 and is seen in fewer than one-third of patients. Namely, types 2 and 3 of SCAD are variants of intramural hematoma. Type 2 has long, smooth stenosis, with the 2a subtype characterized by restoration of blood flow distal to the hematoma. In contrast, type 2b SCAD stenosis is present in the distal segments of the affected vessel. Type 3 is complex to distinguish from a classic focal atherosclerotic lesion. Still, this distinction can be made using intracoronary imaging and other clinical and angiographic features, such as tortuosity of the coronary vessel, presence of small atherosclerotic culprit lesions, and pretest probability (young female, peripartal period, and other SCAD risk factors). SCAD type 4 presents with vessel occlusions that do not meet the criteria for types 1–3, including extensive proximal dissections, hybrid appearances, and diffuse nonfocal narrowing (12).

### Outcomes

The primary endpoint was the occurrence of a MACE, which is a composite of recurrent myocardial infarction, cardiogenic shock, complex ventricular arrhythmias, congestive heart failure, unplanned revascularization (PCI/CABG), stroke, and death (defined as death from any cause). Also, so-called minor adverse events (secondary AE) were followed, and they included chest pain, a visit to the cardiac emergency department, and newly diagnosed atrial fibrillation.

### Statistical analysis

The mean and standard deviation were calculated for normally distributed continuous variables, while median and inter-quartile ranges (IQRs) were obtained for non-normally distributed variables. Counts and proportions were used to describe categorical variables. Univariable and multivariable logistic regression models were used to evaluate the association between pre-specified clinical variables and MACE. For each parameter, odds ratios (ORs), 95% confidence intervals (CIs), and *P*-values were calculated. Pre-specified variables were selected based on prior literature and those that seemed clinically relevant. The recruitment method (prospective vs. retrospective) was not included as a variable in the outcome analysis. All parameters with a *p*-value < 0.05 in the univariable analysis were included in the multivariable analysis. A backward stepwise selection process was used to obtain the final model. Statistical analyses were performed using IBM SPSS Statistics version 26.0, with a two-sided *p*-value < 0.05 considered statistically significant.

### Statistical analysis—modeling strategy

To identify factors associated with the study outcome, a two-step modelling approach was applied. First, univariable analyses were performed for all candidate variables. Variables showing a potential association with the outcome (*p* < 0.10) were considered for inclusion in the multivariable model. Multivariable analysis was performed to identify independent predictors while minimizing the risk of overfitting. The number of variables entered into the model was limited according to the number of outcome events. Before model construction, collinearity between variables was assessed. Only variables that remained significant after adjustment were retained in the final model. Model calibration was evaluated using the Hosmer–Lemeshow goodness-of-fit test. A non-significant result (*p* < 0.05) was considered indicative of adequate model fit.

## Results

In the retrospective part, we included 27 patients, and in the prospective part, we enrolled 96 patients. Table 1 summarizes the baseline demographic, clinical, and echocardiographic characteristics of SCAD patients included in the national registry, both retrospectively and prospectively.

**Table 1:** Baseline characteristics.

Investigated parameter	Additional information	Total *n* = 123	Prospective *n* = 96	Retrospective *n* = 27	*p*-value
Age, years (mean ± SD)		47.84 ± 11.84	47.15 ± 11.07	50.30 ± 14.24	0.224
Sex	Female	105 (85.4)	85 (88.5)	20 (74.1)	
	Male	18 (14.6)	11 (11.5)	7 (25.9)	0.060
Body mass index, kg/m^2^ (mean ± SD)		26.06 ± 5.54	25.60 ± 4.39	26.20 ± 4.51	0.557
Pregnancy or post-partum		12 (9.8)	5 (5.2)	7 (25.9)	**0**.**003**
Number of previous pregnancies		2.46 ± 1.74	2.53 ± 1.64	2.25 ± 2.04	0.580
Hormone replacement therapy or oral contraceptive		11 (8.9)	11 (14.3)	0 (0.0)	0.045
*In vitro* fertilization		6 (4.9)	4 (5.7)	2 (10.0)	0.498
Menopause		35 (28.5)	30 (36.6)	5 (41.7)	0.743
Hypertension		61 (49.6)	46 (48.4)	15 (57.7)	0.402
Diabetes mellitus		7 (5.7)	7 (9.2)	0 (0.0)	0.116
Dyslipidaemia		57 (46.3)	49 (51.6)	8 (29.6)	**0**.**044**
Previous heart diseases		11 (8.9)	10 (10.4)	1 (3.7)	0.280
Cardiac interventions before SCAD		6 (8.6)	6 (8.6)	0 (0.0)	0.151
Previous cerebrovascular diseases		10 (8.1)	8 (9.4)	2 (7.4)	0.890
Cerebral aneurysms		2 (1.6)	2 (2.8)	0 (0.0)	0.211
Sudden cardiac death in the family		11 (8.9)	8 (9.4)	3 (11.1)	0.796
Family history of MI		22 (17.9)	19 (26.4)	3 (12.5)	0.161
Thyroid diseases		13 (10.6)	13 (16.9)	0 (0.0)	**0**.**028**
Hormone replacement therapy in thyroid diseases		11 (8.9)	11 (14.3)	0 (0.0)	**0**.**045**
Known fibromuscular displasia			2 (2.8)	1 (4.2)	0.735
Migraine		12 (9.8)	10 (11.5)	2 (7.7)	0.581
Depression		6 (4.9)	6 (8.5)	0 (0.0)	0.165
Smoking status	Non-smoker	59 (48.0)	46 (47.90)	13 (48.1)	
	Current smoker	51 (41.5)	38 (39.6)	13 (48.1)	
	Ex-smoke	9 (7.3)	8 (8.3)	1 (3.7)	
	Missing	4 (3.3)	4 (4.2)	0 (0.0)	0.549
Type of ACS at presentation	STEMI	84 (68.3)	67 (69.8)	17 (63.0)	
	NSTEMI	39 (31.7)	29 (30.2)	10 (37.0)	0.501
Left ventricular ejection fraction (LVEF)		51.18 ± 10.34	51.72 ± 10.26	49.30 ± 10.62	0.285
LVEF range	<40%	22 (18.2)	16 (17.0)	6 (22.2)	
	40–50%	36 (29.7)	27 (28.7)	9 (33.3)	
	>50%	63 (52.1)	51 (54.3)	12 (44.4)	0.654
Typical symptoms/pain, dyspnoea		96 (78.0)	72 (75.0)	24 (88.8)	0.251
Precipitating factors	unknown	33 (27.0)	28 (29.2)	5 (19.2)	
	mental stress	47 (38.5)	34 (35.4)	13 (50.0)	
	with no known stress	29 (23.8)	25 (26.0)	4 (15.4)	
	physical stress	13 (10.7)	9 (9.4)	4 (15.4)	0.315
Number of previous pregnancies	No	7 (6.7)	4 (4.7)	3 15.0)	
	One	13 (12.4)	9 (10.6)	4 (20.0)	
	Two and more	85 (81.0)	72 (84.7)	13 (65.0)	0.105

SCAD, spontaneous coronary artery dissection; ACS, acute coronary syndrome.

The bolded *p*-values are statistically significant.

The mean age of participants was 47.85 ± 11.84 years, with a predominance of females (85.4%). In terms of clinical presentation, the majority of patients presented with ST-segment elevation myocardial infarction (STEMI).

Typical symptoms for ACS (chest pain and dyspnoea) were reported in nearly all patients in both groups. Overall, mental stress was the most frequently reported precipitating factor.

Approximately 10% of patients were either pregnant or in the postpartum period, and the average number of previous pregnancies was 2.46 ± 1.74. There were 162 pregnancies, with 96 live births and 66 failed pregnancies. Menopause was reported in 28.5% of women.

Regarding cardiovascular risk factors, nearly half of the patients had hypertension (49.6%) or dyslipidemia (46.3%), while diabetes mellitus was less common at 5.7%. Other comorbidities included migraine and depression. Known fibromuscular dysplasia was identified in 2.4% of cases. Notably, dyslipidemia was more prevalent among prospectively enrolled patients. At the same time, no significant differences were observed between groups for other cardiovascular risk factors, reproductive history variables, or comorbidities, including arterial hypertension, diabetes, migraine, or depression.

52.1% of patients had a left ventricular ejection fraction (LVEF) above 50%, while 18.2% had an LVEF below 40%. The distribution across LVEF categories (<40%, 40%–50%, >50%) was also similar between the groups.

The angiographic characteristics are given in Table 2. The therapeutic approach (PCI/POBA vs. medication-only) did not differ significantly between groups. Intracoronary imaging (OCT/IVUS) was performed in 32 patients. In 10 (8.1%) patients, SCAD propagation was seen during PCI.

**Table 2:** Coronary angiographic findings.

Angiographic characteristic	Additional information	Total *n* = 123	Prospective *n* = 96	Retrospective *n* = 27	*p*-value
Diameter of the vessel (cm)		2.88 ± 0.75	2.94 ± 0.79	2.68 ± 0.56	0.195
The length of the lesson (mm)		34.30 ± 18.42	37.38 ± 20.07	26.21 ± 9.52	**0**.**023**
>50% stenosis		74 (60.1)	54 (56.3)	20 (74.0)	0.731
Coronary artery affected	LM	5 (4.1)	5 (5.2)	0 (0.0)	
	LAD	69 (56.1)	53 (55.2)	16 (59.2)	
	LCx	1 (0.8)	1 (1.04)	0 (0.0)	
	RCA	11 (8.9)	6 (6.2)	5 (18.5)	
	RI	4 (3.3)	3 (3.1)	1 (3.7)	
	DG	2 (1.6)	0 (0.0)	2 (7.4)	
	OM	5 (4.1)	4 (4.2)	1 (3.7)	
	More vessels	1 (0.8)	1 (1.04)	0 (0.0)	
	PDA	1 (0.8)	1 (1.04)	0 (0.0)	0.189
Type of SCAD	Type 1	25 (21.9)	21 (24.1)	4 (14.8)	
	Type 2A	48 (42.1)	36 (41.4)	12 (44.4)	
	Type 2B	16 (14.0)	12 (13.8)	4 (14.8)	
	Type 3	7 (6.1)	5 (5.7)	2 (7.4)	
	Type 4	18 (15.8)	13 (17.9)	5 (18.5)	0.888
Location of SCAD in the vessel	Proximal	1 (3.1)	1 (4.8)	0 (0.0)	
	Mid	6 (18.8)	2 (9.5)	4 (36.4)	
	Distal	7 (21.9)	5 (23.8)	2 (18.2)	
	More	18 (56.3)	13 (61.9)	5 (455)	0.290
Number of SCAD arteries	One	93 (93.9)	68 (91.9)	25 (100.0)	
	Two	5 (5.1)	5 (6.8)	0 (0.0)	
	Three	1 (1.0)	1 (1.4)	0 (0.0)	0.340
Type of therapy	PCI	36 (29.2)	25 (26.1)	11 (40.7)	0.147
	POBA	31 (25.2	26 (27.1)	5 (18.5)	0.284
	Drugs	60 (48.7)	47 (48.9)	13 (48.1)	0.466
Number of stents implanted	One	18 (54.5)	12 (52.2)	6 (60.0)	
	Two	4 (12.1)	2 (87)	2 (20.0)	
	Three	7 (21.2)	6 (26.1)	1 (10.0)	
	Four	4 (12.2)	3 (13.0)	1 (10.0)	0.633
Angiographic approach	Radial	91 (74.0)	70 (72.9)	21 (77.8)	
	Femoral	32 (26.0)	26 (27.1)	6 (22.2)	0.611
Complications	Recurrent MI	7 (5.7)	6 (6.3)	1 (3.7)	**0**.**021**
	Hemodynamic instability	10 (8.1)	8 (8.3)	2 (7.4)	0.255
	Malignant arrhythmias	14 (11.4)	12 (12.5)	2 (7.4)	0.195
	Congestive heart failure	11 (8.9)	10 (10.4)	1 (3.7)	0.113
	Unplanned revascularization	8 (6.5)	8 (8.3)	0 (0.0)	0.120
	Stroke	1 (0.8)	0 (0.0)	1 (3.7)	0.250
	Resolution of ST-segment elevation	88 (71.5)	67 (69.8)	21 (77.8)	0.599
TIMI flows at the beginning	TIMI 0	17 (13.8)	12 (13.0)	5 (18.5)	
	TIMI 1	25 (20.3)	19 (20.7)	6 (22.0)	
	TIMI 2	15 (12.2)	9 (9.8)	6 (22.2)	
	TIMI 3	62 (50.4)	52 (56.5)	10 (37.0)	0.208
TIMI flows at the end of the procedure	TIMI 0	6 (4.9)	4 (6.3)	2 (8.3)	
	TIMI 1	6 (4.9)	4 (6.3)	2 (8.3)	
	TIMI 2	10 (8.1)	8 (12.5)	2 (8.3)	
	TIMI 3	66 (53.7)	48 (75.0)	18 (75.0)	0.921

LAD, left anterior descending artery; LM, left main coronary artery; LCx, left circumflex artery; RCA, right coronary artery; PDA, posterior descending artery; OM, obtuse marginal branch DG, diagonal branch; RI, ramus intermedius.

The bolded *p*-values are statistically significant.

After 30 days, 23 patients (62.2%) without initial PCI/POBA and 14 (37.8%) with prior PCI/POBA underwent coronary angiography. Of these, two patients from each group underwent repeated PCI.

Table 3 presents data on the antiplatelet and additional pharmacological therapies administered to two patient groups. Regarding antiplatelet agents, aspirin was the most commonly prescribed drug, followed by ticagrelor and clopidogrel.

**Table 3:** Medication therapy.

Investigated parameter	Additional information	Total	Prospective *n* = 96	Retrospective *n* = 27	*p*-value
Type of therapy	SAPT	16 (13)	13 (13.5)	3 (11.1)	
	DAPT	107 (87)	83 (86.4)	24 (88.9)	0.762
LMWH		70 (56.9)	53 (55.2)	17 (62.9)	0.555
Aspirin		79 (64.2)	66 (68.7)	13 (48.1)	**<0**.**001**
Clopidogrel		12 (9.8)	10 (10.4)	2 (7.4)	0.572
Ticagrelor		16 (13.0)	11 (11.4)	5 (18.5)	0.393
Statin		28 (22.8)	17 (17.7)	11 (40.7)	**0**.**014**

SAPT, single antiplatelet therapy; DAPT, dual antiplatelet therapy; LMWH, low molecular weight heparin.

The bolded *p*-values are statistically significant.

There was a highly significant difference in aspirin use, with the prospective group showing much higher use than the retrospective group. Statin therapy was significantly more frequent in the retrospective group.

Table 4 presents the incidence of major adverse cardiovascular events (MACE).

**Table 4:** Major adverse cardiovascular events (MACE), minor adverse events (secondary AE), and death.

Investigated parameter	Total *n* = 123	Prospective *n* = 96	Retrospective *n* = 27	*p*-value
MACE during hospitalization	29 (23.6)	24 (30.4)	5 (20.8)	0.362
Death during hospitalization	10 (8.1)	8 (8.6)	2 (7.4)	0.843
MACE after 30 days	10 (8.1)	6 (8.5)	4 (15.4)	0.320
Secondary AE after 30 days	15 (12.2)	12 (18.2)	3 (11.5)	0.437
MACE after one year	5 (8.2)	4 (9.8)	1 (5.0)	0.525
Secondary AE after one year	5 (8.2)	4 (9.8)	1 (5.0)	0.525

After 30 days of follow-up, one patient died, 1 had a stroke, three were hospitalized due to congestive heart failure, 3 had myocardial reinfarction, and 2 of them needed urgent revascularization, one with PCI and one with CABG.

## Discussion

Results from the Serbian (RS) SCAD Registry demonstrate a notably higher prevalence of traditional cardiovascular risk factors, such as arterial hypertension, dyslipidemia, and active smoking, compared to many published Western registries. The PCI utilization rate in our cohort is substantially higher than reported in contemporary Western registries. Further, in-hospital mortality and MACE occurred more frequently than in most major published registries (13–15). Multivessel SCAD involvement was independently associated with a higher intrahospital mortality rate. These findings suggest that the Serbian SCAD population has a distinct phenotype or that management strategies should be reassessed.

As in other registries, the majority of patients were female, which aligns with broader global trends, as evidenced by data from European and North American registries, highlighting a consistent demographic pattern in SCAD presentation (16). The mean age of 47.8 ± 11.84 years is comparable to that reported in Western registries, which typically report mean ages of 48–52 years (6).

Chest pain or chest discomfort is by far the most common presenting symptom in SCAD cohorts, reported in 90%–96% of patients, consistent with our data (17). STEMI was the predominant presentation (68.3%), somewhat higher than in some Western cohorts but consistent with the heterogeneous presentation spectrum of SCAD (6). Despite STEMI being a common presentation, the majority of SCAD patients have a preserved or mildly reduced left ventricular ejection fraction (LVEF), as was the case in our patients (18).

Emotional stress was the most frequent in both of our patient groups and was the most frequently identified precipitating factor (38.5%), consistent with international SCAD literature, which recognizes emotional stress as a prominent precipitating factor, particularly in women (2, 19).

Hypertension (49.6%) in our cohort substantially exceeds the 33.9% reported in the prospective Canadian SCAD cohort and the 37% documented in the Spanish SR-SCAD registry (20, 21). In the analysis of Alfonso et al.'s analysis on 318 SCAD patients, hypertensive patients were significantly older, had a higher prevalence of dyslipidemia and diabetes, and they had more severe angiographic lesions with higher rates of multivessel involvement and coronary ectasia. During revascularization attempts, hypertensive patients had significantly lower procedural success and higher procedure-related complications (21). Our hypertensive patients likely constitute a higher-risk population requiring particularly judicious decision-making regarding revascularization.

Dyslipidemia in our registry (46.3%) similarly exceeds typical Western registry rates (26%–35%), and smoking prevalence (41.5%) is notably higher compared to Canadian (33%) and Spanish (25%) cohorts.

This distinctive risk factor profile is not a reflection of older age, as our mean age (47.8 years) matches Western registries (20, 21). Notably, it suggests either true population differences in cardiovascular risk factor prevalence in SCAD patients in this geographic region, differences in screening practices, or potentially in treatment. These findings challenge the traditional characterization of SCAD as affecting predominantly “healthy” women without atherosclerotic risk factors, aligning with more contemporary registry data indicating that SCAD and traditional risk factors are increasingly recognized as coexisting entities.

Pregnancy-associated SCAD affects approximately 1.81 per 100,000 pregnancies. Approximately 10% of our patients were either pregnant or in the postpartum period, which was significantly more common in the retrospective group. Conversely, in the Australia-New Zealand (ANZ) SCAD registry, peripartum SCAD was more common in the prospective group. This mismatch between cohorts could be explained by differences in the methodologies used for retrospective and prospective case identification. Prospective cohorts typically enroll patients at the time of acute presentation, and pregnancy-associated SCAD occurs between 2 and 32 weeks after delivery, meaning many postpartum cases may present outside standard prospective enrollment windows in some studies (3, 22, 23).

8.9% of included females were taking sex hormone therapy or were on *in vitro* fertilization treatment. These underscore the influence of hormonal fluctuations on SCAD development (24).

A high percentage of our patients were postmenopausal women (28.5%). Postmenopausal women with SCAD exhibit a distinct risk factor profile, typically being older and having a higher prevalence of traditional risk factors compared to premenopausal women, yet paradoxically presenting less frequently with STEMI. The relationship between estrogen deficiency and SCAD differs from the typical atherosclerotic disease pattern in postmenopausal women, suggesting distinct pathophysiological mechanisms. This observation aligns with growing recognition in the literature that sex-specific risk factors, including menopause and reproductive history, play important roles in SCAD pathophysiology (25).

We found a high incidence of failed pregnancies among female patients. SCAD patients frequently have connective tissue disorders and vasculopathies that predispose them to both SCAD and potentially to placental vascular compromise (26). Also, hormonal changes that precipitate SCAD may also contribute to placental vascular dysfunction (2, 27, 28).

The most commonly affected artery in our cohort was the left anterior descending coronary artery (LAD) (56.1%), consistent with published SCAD registries, which report LAD involvement ranging from 50% to 68%. Type 2a SCAD was the most common pattern (42.1%), a finding consistent with the distribution in Western registries. The predominance of type 2a SCAD in the distal LAD represents a common phenotype across diverse geographic populations, suggesting shared pathophysiological mechanisms (15, 29, 30). Intracoronary imaging (OCT/IVUS) was performed in 32 patients (26%), raising the possibility of SCAD subtype misclassification, especially for type 3 SCAD lesions, which can mimic atherosclerotic disease. Coronary computed tomography angiography (CCTA) has emerged as a valuable non-invasive diagnostic modality for SCAD, particularly in hemodynamically stable patients, and may offer safety advantages while allowing assessment of extracoronary arteriopathies (31).

The most notable difference between our cohort and Western registries is the rate of PCI utilization. Our 51.3% PCI rate contrasts sharply with the Spanish registry (22%), Canadian cohort (14.1%), and typical contemporary rates from other registries (15%–25%) (6, 32). Modern practice has evolved significantly toward more conservative management (33, 34).

We included patients from 2014 to 2024, which may partially explain the overuse of PCI and underutilization of contemporary guidelines. The high PCI utilization in our cohort may reflect operator-dependent decision-making, limited availability of formal guidelines, reliance on expert consensus, and insufficient training in SCAD-specific diagnosis and management.

Our patients received more aggressive antiplatelet therapy. Most patients in both our groups received DAPT (87%), compared with those in Western cohorts (65%–70%) (13–15). The European multicenter DISCO registry found that, among conservatively managed SCAD patients, those treated with DAPT had a significantly higher rate of MACE at 1 year compared to those on SAPT (35). Similar findings were reported from the Spanish SR-SCAD registry and the Australian-New Zealand SCAD cohort (22).

23.6% of patients experienced MACE during hospitalization, and 8.1% of patients died during hospitalization. The in-hospital MACE rate is notably higher than in most large prospective SCAD registries. The Canadian cohort, with a similar prospective design and 750 patients, reported in-hospital MACE of only 8.8% and mortality of approximately 1.3%. Even the Spanish registry reported in-hospital MACE of 8–11.7% (14, 15, 32). This difference in MACE incidence cannot be entirely explained by our higher PCI rate and DAPT use, suggesting that additional factors, including health system and patient characteristics, warrant further investigation.

Due to the limited number of MACE events, the regression analyses are likely underpowered and should be interpreted with caution, serving a descriptive purpose primarily. Accordingly, these results are presented in the Supplementary Material (Tables S5–S8) to ensure transparency.

The one-year MACE rate of 8.2% in our registry appears lower than long-term MACE reported in major international SCAD registries (Canadian: 14%; Spanish: 13%; ANZ: 7%). However, direct comparison is limited by differences in follow-up duration, sample size, and potentially baseline patient characteristics. An extended follow-up of our cohort and a head-to-head comparison of registry methodologies will clarify whether this difference reflects better long-term prognosis or methodological variation (14, 15, 23).

## Study limitations

This study has several limitations. First, as an observational, non-randomized registry, causal relationships between management strategies and outcomes cannot be established, and local practice rather than standardized protocols influenced treatment decisions. Second, the inclusion of both retrospective and prospective cohorts introduces potential selection and information bias, with incomplete documentation of some clinical, hormonal, and psychosocial variables in the retrospective arm. Third, the moderate sample size and low number of clinical events, particularly at 30 days and 1 year, limit statistical power and yield wide confidence intervals in multivariable analyses; therefore, observed associations should be interpreted cautiously and are presented in the Supplementary Material. Intracoronary imaging was performed in a minority of patients, raising the possibility of SCAD subtype misclassification, especially for type 3 SCAD. Systematic screening for extracoronary arteriopathies, including fibromuscular dysplasia, was not performed, likely resulting in an underestimate of their prevalence.

Finally, variability in operator experience and the absence of standardized SCAD-specific protocols may have influenced treatment decisions and further outcomes. The lack of structured guidelines and the reliance on experts’ consensus for SCAD treatment were the strongest motivations for starting the Registry.

## Conclusion

Published SCAD registries have largely originated from high-income Western regions, including North America, Europe, and Australia/New Zealand. The Serbian SCAD Registry offers novel insights into an Eastern European transitional population and reveals important differences compared with established Western cohorts. Although the mean age (47.8 years) and female predominance (85.4%) are consistent with global SCAD demographics, our cohort demonstrates a substantially higher burden of traditional cardiovascular risk factors—hypertension, dyslipidemia, and smoking—than most previously published registries. These findings challenge the traditional perception of SCAD as a condition affecting otherwise “healthy” women and highlight the importance of comprehensive cardiovascular risk assessment and management in this population.

The in-hospital MACE rate (23.6%) and mortality rate (8.1%) observed in our cohort are markedly higher than those reported in major prospective Western registries. While these differences may only partially be explained by more frequent use of PCI and DAPT than in other registries, which is associated with the worse outcomes in SCAD patients, they likely also point to broader system-level and practice-related differences that warrant further investigation (10, 35, 36).

Optimizing outcomes in SCAD requires timely and accurate diagnosis, improved clinician awareness, and strict adherence to contemporary international recommendations that prioritize conservative therapy whenever feasible. In parallel, patients should undergo systematic evaluation for predisposing arteriopathies and receive structured, evidence-based management of cardiovascular risk factors.

Finally, comparative data from diverse geographic regions are essential to determine whether the differences observed in this registry reflect true epidemiologic variation or modifiable healthcare system factors. Addressing potential gaps through education, guideline dissemination, and standardized management pathways may represent a critical step toward improving SCAD outcomes globally.

## Data Availability

The raw data supporting the conclusions of this article will be made available by the authors, without undue reservation.

## References

[1] Antonutti M Baldan F Lanera C Spedicato L Zanuttini D Bisceglia T Spontaneous coronary artery dissection: role of prognostic markers and relationship with genetic analysis. Int J Cardiol. (2021) 326:19–29. 10.1016/j.ijcard.2020.10.040 33190788

[2] Stanojevic D Apostolovic S Kostic T Mitov V Kutlesic-Kurtovic D Kovacevic M A review of the risk and precipitating factors for spontaneous coronary artery dissection. Front Cardiovasc Med. (2023) 10:1273301. 10.3389/fcvm.2023.1273301 38169687 PMC10758453

[3] Apostolović S Ignjatović A Stanojević D Radojković DD Nikolić M Milošević J Spontaneous coronary artery dissection in women in the generative period: clinical characteristics, treatment, and outcome-a systematic review and meta-analysis. Front Cardiovasc Med. (2024) 11:1277604. 10.3389/fcvm.2024.1277604 38390446 PMC10882101

[4] Djokovic A Krljanac G Matic P Zivic R Djulejic V Marjanovic Haljilji M Pathophysiology of spontaneous coronary artery dissection: hematoma, not thrombus. Front Cardiovasc Med. (2023) 10:1260478. 10.3389/fcvm.2023.1260478 37928766 PMC10623160

[5] Krljanac G Apostolovic S Mehmedbegovic Z Nedeljkovic-Arsenovic O Maksimovic R Ilic I Chronic or changeable infarct size after spontaneous coronary artery dissection. Diagnostics. (2023) 13(9):1518. 10.3390/diagnostics13091518 37174911 PMC10177350

[6] Hayes SN Kim ESH Saw J Adlam D Arslanian-Engoren C Economy KE Spontaneous coronary artery dissection: current state of the science: a scientific statement from the American Heart Association. Circulation. (2018) 137(19):e523–57. 10.1161/CIR.0000000000000564 29472380 PMC5957087

[7] Mori R Macaya F Giacobbe F Salinas P Pavani M Boi A Clinical outcomes by angiographic type of spontaneous coronary artery dissection. EuroIntervention. (2021) 17(6):516–24. 10.4244/EIJ-D-20-01275 33650491 PMC9724881

[8] Kądziela J Kochman J Grygier M Michałowska I Tomaniak M Wojakowski W The diagnosis and management of spontaneous coronary artery dissection—expert opinion of the association of cardiovascular interventions (ACVI) of Polish cardiac society. Kardiol Pol. (2021) 79(7–8):930–43. 10.33963/KP.a2021.0068 34292564

[9] Agwuegbo CC Ahmed EN Olumuyide E Moideen Sheriff S Waduge SA. Spontaneous coronary artery dissection: an updated comprehensive review. Cureus. (2024) 16(2):e55106. 10.7759/cureus.55106 38558647 PMC10979520

[10] Petrović M Miljković T Ilić A Kovačević M Čanković M Dabović D Management and outcomes of spontaneous coronary artery dissection: a systematic review of the literature. Front Cardiovasc Med. (2024) 11:1276521. 10.3389/fcvm.2024.1276521 38298759 PMC10829101

[11] Cader FA Banerjee S Gulati M. Sex differences in acute coronary syndromes: a global perspective. J Cardiovasc Dev Dis. (2022) 9(8):239. 10.3390/jcdd9080239 36005403 PMC9409655

[12] Adlam D Tweet MS Gulati R Kotecha D Rao P Moss AJ Spontaneous coronary artery dissection: pitfalls of angiographic diagnosis and an approach to ambiguous cases. JACC Cardiovasc Interv. (2021) 14(16):1743–56. 10.1016/j.jcin.2021.06.027 34412792 PMC8383825

[13] García-Guimaraes M Bastante T Macaya F Roura G Sanz R Barahona Alvarado JC Spontaneous coronary artery dissection in Spain: clinical and angiographic characteristics, management, and in-hospital events. Rev Esp Cardiol. (2021) 74(1):15–23. (In English, Spanish). 10.1016/j.rec.2020.04.002 32418854

[14] Kim SK Wing-Lun E Chandrasekhar J Puri A Burgess S Ford TJ The Australian New Zealand spontaneous coronary artery dissection (ANZ-SCAD) registry—a multi-centre cohort study: protocol, background and significance. Heart Lung Circ. (2022) 31(12):1612–8. 10.1016/j.hlc.2022.08.018 36180304

[15] Saw J Starovoytov A Aymong E Inohara T Alfadhel M McAlister C Canadian Spontaneous coronary artery dissection cohort study: 3-year outcomes. J Am Coll Cardiol. (2022) 80(17):1585–97. 10.1016/j.jacc.2022.08.759 36265953

[16] Daoulah A Al-Faifi SM Alhamid S Youssef AA Alshehri M Al-Murayeh M Spontaneous coronary artery dissection in the gulf: g-SCAD registry. Angiology. (2021) 72(1):32–43. 10.1177/0003319720946974 32787614

[17] Luong C Starovoytov A Heydari M Sedlak T Aymong E Saw J. Clinical presentation of patients with spontaneous coronary artery dissection. Catheter Cardiovasc Interv. (2017) 89(7):1149–54. 10.1002/ccd.26977 28244197

[18] Al-Hussaini A Abdelaty AMSEK Gulsin GS Arnold JR Garcia-Guimaraes M Premawardhana D Chronic infarct size after spontaneous coronary artery dissection: implications for pathophysiology and clinical management. Eur Heart J. (2020) 41(23):2197–205. 10.1093/eurheartj/ehz895 31898721 PMC7299635

[19] Saw J Starovoytov A Humphries K Sheth T So D Minhas K Canadian Spontaneous coronary artery dissection cohort study: in-hospital and 30-day outcomes. Eur Heart J. (2019) 40(15):1188–97. 10.1093/eurheartj/ehz007 30698711 PMC6462308

[20] Pabst F Miekisch W Fuchs P Kischkel S Schubert JK. Monitoring of oxidative and metabolic stress during cardiac surgery by means of breath biomarkers: an observational study. J Cardiothorac Surg. (2007) 2:37. 10.1186/1749-8090-2-37 17877828 PMC2100047

[21] Alfonso F García-Guimaraes M Alvarado T Sanz-Ruiz R Roura G Amat-Santos IJ Clinical implications of arterial hypertension in patients with spontaneous coronary artery dissection. Coron Artery Dis. (2022) 33(2):75–80. 10.1097/MCA.0000000000001043 33878074

[22] Dang QM Psaltis PJ Burgess S Chandrasekhar J Mukherjee S Kritharides L The Australian-New Zealand spontaneous coronary artery dissection cohort study: predictors of major adverse cardiovascular events and recurrence. Eur Heart J. (2025) 46(21):2012–23. 10.1093/eurheartj/ehaf097 40049585 PMC12127729

[23] Tweet MS Hayes SN Gulati R Rose CH Best PJ. Pregnancy after spontaneous coronary artery dissection: a case series. Ann Intern Med. (2015) 162(8):598–600. 10.7326/L14-0446 25894037

[24] Kalkman DN Vink AS Beijk MAM van den Born BH Ten Berg JM Arslan F Spontaneous coronary artery dissection: dissecting an underdiagnosed problem. Neth Heart J. (2025) 33(12):385–94. 10.1007/s12471-025-01992-x 41148429 PMC12638533

[25] Díez-Villanueva P García-Guimaraes MM Macaya F Masotti M Nogales JM Jimenez-Kockar M Spontaneous coronary artery dissection and menopause. Am J Cardiol. (2021) 148:53–9. 10.1016/j.amjcard.2021.02.007 33617813

[26] Tarr I Hesselson S Iismaa SE Rath E Monger S Troup M Exploring the genetic architecture of spontaneous coronary artery dissection using whole-genome sequencing. Circ Genom Precis Med. (2022) 15(4):e003527. 10.1161/CIRCGEN.121.003527 35583931 PMC9388555

[27] Carss KJ Baranowska AA Armisen J Webb TR Hamby SE Premawardhana D Spontaneous coronary artery dissection: insights on rare genetic variation from genome sequencing. Circ Genom Precis Med. (2020) 13(6):e003030. 10.1161/CIRCGEN.120.003030 33125268 PMC7748045

[28] Huart J Stoenoiu MS Zedde M Pascarella R Adlam D Persu A. From fibromuscular dysplasia to arterial dissection and back. Am J Hypertens. (2023) 36(11):573–85. 10.1093/ajh/hpad056 37379454

[29] Maričić B Perišić Z Kostić T Božinović N Petrović M Čanković M An analysis of published cases of cutting balloon use in spontaneous coronary artery dissection. Front Cardiovasc Med. (2023) 10:1270530. 10.3389/fcvm.2023.1270530 38028445 PMC10666782

[30] Garcia-Guimaraes M Masotti M Sanz-Ruiz R Macaya F Roura G Nogales JM Spanish Registry on SCAD investigators. Clinical outcomes in spontaneous coronary artery dissection. Heart. (2022) 108(19):1530–8. 10.1136/heartjnl-2022-320830 35410894

[31] Pergola V Continisio S Mantovani F Motta R Mattesi G Marrazzo G Spontaneous coronary artery dissection: the emerging role of coronary computed tomography. Eur Heart J Cardiovasc Imaging. (2023) 24(7):839–50. 10.1093/ehjci/jead060 37082977

[32] Adlam D Alfonso F Maas A Vrints C, Writing Committee, Al-Hussaini A Writing committee, European society of cardiology, acute cardiovascular care association, SCAD study group: a position paper on spontaneous coronary artery dissection. Eur Heart J. (2018) 39(36):3353–68. 10.1093/eurheartj/ehy080 29481627 PMC6148526

[33] Krittanawong C Castillo Rodriguez B Ang SP Qadeer YK Wang Z Alam M Conservative approach versus percutaneous coronary intervention in patients with spontaneous coronary artery dissection from a national population-based cohort study. Rev Cardiovasc Med. (2024) 25(11):404. 10.31083/j.rcm2511404 39618857 PMC11607482

[34] Feldbaum E Thompson EW Cook TS Sanghavi M Wilensky RL Fiorilli PN Management of spontaneous coronary artery dissection: trends over time. Vasc Med. (2023) 28(2):131–8. 10.1177/1358863X231155305 37025021 PMC10084514

[35] Benenati S Giacobbe F Zingarelli A Macaya F Biolè C Rossi A Interventional versus conservative strategy in patients with spontaneous coronary artery dissections: insights from DISCO registry. Circ Cardiovasc Interv. (2023) 16(6):e012780. 10.1161/CIRCINTERVENTIONS.122.012780 37259861 PMC10810347

[36] Maremmani M La Porta Y Centola M Spina M Rogacka R Mollichelli N Management and outcome of spontaneous coronary artery dissections: a single– centre experience. Eur Heart J Suppl. (2025) 27(Supplement_5):suaf076–187. 10.1093/eurheartjsupp/suaf076.187

