# Supplementary material for: Spontaneous coronary artery dissection in an underrepresented region: insights from the Serbian (RS) SCAD registry
Source: Front Cardiovasc Med. 2026 May 11;13:1795347. doi: 10.3389/fcvm.2026.1795347 (PMC13198987; doi:10.3389/fcvm.2026.1795347)
Supplement: Supplementary file 1 [file Datasheet1.pdf]

Supplementary material – univariate and multivariate analysis

In-hospital MACE

In multivariable analysis, statin therapy and SCAD presentation (STEMI) remained independent predictors of in-hospital MACE. Cardiac interventions before SCAD showed a strong association but did not reach statistical significance (p=0.055) (Table 5).

In-hospital death

In the univariable logistic regression analysis for in-hospital mortality, multivessel SCAD, POBA, and LMWH therapy were significantly associated with the outcome. In the multivariable model, only multivessel SCAD remained an independent predictor of in-hospital death (p=0.010) (Table 6).

MACE 30 days after hospitalization

Univariable logistic regression analysis did not identify any variable as a statistically significant predictor of MACE occurring within 30 days after hospitalization. Depression, stent implantation, and the presence of cerebral aneurysms demonstrated borderline significance. Female sex, STEMI presentation, SCAD-affected arterial segment (proximal, mid, or distal), and PCI were not significantly associated with 30-day MACE (Table 7).

Minor AE 30 days after hospitalization

In the multivariable model, age (OR 1.11, p=0.021) and migraine (OR 7.31, p=0.012) remained independent predictors of minor adverse events 30 days post-hospitalization (Table 8).

1-year prognosis

In the univariate logistic regression analysis of potential predictors of MACE at 1 year after hospitalization, most variables did not reach statistical significance. Among all examined predictors, depression was the only variable significantly associated with increased odds of one-year MACE (OR = 23.00, 95% CI: 1.023–516.933, p=0.048). However, the wide confidence interval indicates that the small sample size and limited number of events prevent accurate measurement of the effect sizes.

Table 5. Univariate and multivariate logistic regression analysis for intrahospital MACE

	Univariate		Multivariate	
**Variable**	**OR (95% CI)**	**p**	**OR (95% CI)**	**p**
Sex (female vs male)	1.200 (0.301–4.787)	0.796	–	–
Age (per year)	1.000 (1.000–1.001)	0.531	–	–
BMI (kg/m²)	1.152 (0.925–1.435)	0.206	–	–
Pregnancy/postpartum	0.165 (0.020–1.348)	0.093	–	–
Hypertension	1.133 (0.473–2.713)	0.779	–	–
Diabetes mellitus	2.217 (0.461–10.656)	0.320	–	–
Dyslipidemia	0.582 (0.244–1.391)	0.224	–	–

Previous heart disease	3.286 (0.867–12.453)	0.080	–	–
Cardiac interventions before SCAD	6.190 (1.057–36.243)	**0.043**	9.170 (0.954–88.165)	0.055
Multivessel SCAD (≥2 arteries)	5.849 (1.104–30.974)	**0.038**	–	–
STEMI	0.137 (0.030–0.621)	**0.010**	0.056 (0.006–0.491)	**0.009**
Statin therapy	7.564 (2.718–21.047)	**<0.001**	11.114 (3.253–37.977)	**<0.001**
PCI total	0.943 (0.362–2.461)	0.905	–	–
POBA	2.579 (0.962–6.916)	0.060	–	–
Stent implantation	2.143 (0.835–5.499)	0.113	–	–

Segment of SCAD artery affected (proximal, mid, distal)	0.664(0.244-1.805)	0.422		
Depression	1.292 (0.222–7.519)	0.776	–	–
Migraine	0.603 (0.117–3.100)	0.545	–	–
Known fibromuscular dysplasia	2.345 (0.690–7.966)	0.172	–	–

Table 6. Univariate and multivariate logistic regression analysis for intrahospital death

	Univariate			Multivariate		
**Variable**	OR	95%CI	p	OR	95%CI	p
**Sex**	1.000	0.999-1.001	0.876	—	—	—
**Age**	0.357	0.083-1.534	0.166	—	—	—
**Multivessel SCAD**	12.000	1.941-74.184	**0.008***	**15.444**	1.913-124.644	**0.010***
**POBA**	4.565	1.133-18.390	**0.033***	1.344	0.092-19.600	0.829
**LMWH**	0.154	0.031-0.762	**0.022***	0.122	0.006-2.420	0.122
**FMD**	3.335	0.909-12.245	0.069	—	—	—
**Stent implantation**	3.208	0.807-12.763	0.098	—	—	—
**Use of statins**	3.660	0.576-23.248	0.169	—	—	—
**Dyslipidemia**	0.259	0.053-1.275	0.097	—	—	—

SCAD -spontaneous coronary artery dissection, POBA -plain old balloon angioplasty, LMWH -low molecular weight heparin, FMD – fibromuscular dysplasia

Table 7. Univariate and multivariate logistic regression analysis for 30-day MACE

	Univariate		
**Variable**	**OR**	**95% CI**	**p**
**Female**	0.338	0.076-1.503	0.154
**Age**	1.028	0.968-1.092	0.372
**Depression**	5.250	0.759-36.334	0.093
**STEMI**	0.211	0.026-1.746	0.149
**Segment of SCAD artery affected (proximal, mid, distal)**	0.213	0.030-1.523	0.123
**Cerebral aneurysms**	1.988	0.890-4.438	0.094
**STENT implantation**	3.580	0.881-14.538	0.075
**PCI total (stent implantation and POBA)**	2.955	0.781-11.177	0.111

PCI -percutaneous coronary intervention

Table 8. Univariate and multivariate logistic regression analysis for 30-day miniAE

	**Univariate**			**Multivariate**		
**Variable**	**OR**	**95% CI**	**p**	**OR**	**95% CI**	**p**
**Female**	2.585	0.310-21.536	0.380			
**Age**	0.898	0.837-0.964	**0.003**	1.111	1.016-1.214	**0.021**
**Depression**	11.625	1.679-80.474	**0.013**	-	-	-
**Migraine**	6.979	1.730-28.161	**0.006**	7.308	1.535-34.798	**0.012**
**Hypertension**	0.271	0.079-0.928	**0.038**	0.943	0.196-3.960	0.869
**STENT implantation**	2.245	0.676-7.459	0.187	-	-	-
**STEMI**	0.756	0.219-2.613	0.659	-	-	-
**Segment of SCAD artery affected (proximal, mid, distal)**	1.130	0.302-4.226	0.856	-	-	-
